# Exploring phytochemicals and pharmacological properties of *Populus × tomentiglandulosa*

**DOI:** 10.3389/fphar.2024.1406623

**Published:** 2024-08-28

**Authors:** Hak-Dong Lee, Ji Hyun Kim, Jong Hee Choi, Ki Hyun Kim, Jajung Ku, Kyung Choi, Hyun Young Kim, Sanghyun Lee, Ik-Hyun Cho

**Affiliations:** ^1^ Department of Plant Science and Technology, Chung-Ang University, Anseong, Republic of Korea; ^2^ Natural Product Institute of Science and Technology, Anseong, Republic of Korea; ^3^ Department of Food Science, Gyeongsang National University, Jinju, Republic of Korea; ^4^ Department of Convergence Medical Science, College of Korean Medicine, Kyung Hee University, Seoul, Republic of Korea; ^5^ School of Pharmacy, Sungkyunkwan University, Suwon, Republic of Korea; ^6^ Department of Forest Bioresources, National Institute of Forest Science, Suwon, Republic of Korea; ^7^ Garden and Plant Resources Division, Korea National Arboretum, Pocheon, Republic of Korea

**Keywords:** *Populus × tomentiglandulosa*, biochemical profiling, free radical, anti-inflammation, phytomedicine

## Abstract

*Populus × tomentiglandulosa* (PT), a tree endemic to Korea, shows promising potential as a natural therapeutic agent owing to its potent anti-inflammatory properties. However, the isolation and analysis of phytochemical compounds in PT and related species remains underexplored. Therefore, this study aims to investigate the biochemical profile of PT and evaluate its extracts and fractions for anti-inflammatory activities. Nine compounds were isolated, including two novel flavonoids (luteolin 7-O-*β*-d-glucuronide butyl ester and chrysoeriol 7-O-*β*-d-glucuronide butyl ester) from the Salicaceae family for the first time. The ethyl acetate fraction exhibited significant radical scavenging activity against various radicals, including DPPH, ABTS^+^, ^•^OH, and O_2_^–^ radicals. PT extracts and the ethyl acetate fraction showed minimal cytotoxicity in Raw 264.7 macrophages at concentrations below 500 and 100 μg/mL, respectively. Furthermore, PT extracts and fractions significantly suppressed the protein expression of proinflammatory mediators (iNOS and IL-6) in LPS-stimulated Raw 264.7 macrophages, highlighting their potent anti-inflammatory effects. These findings suggest that PT holds promise as a valuable natural therapeutic intervention for various oxidative stress and inflammation-related disorders, underscoring the need for further exploration of its clinical applications.

## 1 Introduction

The term “reactive oxygen species” (ROS) includes oxygen free radicals such as the superoxide anion radical (O_2_^–^), singlet oxygen (^1^O_2_), hydroxyl radical (^·^OH), and perhydroxyl radical (HO_2_·) (Phaniendra et al., 2015). ROS are naturally and continually generated through physiological processes in the human body. Free radicals have beneficial effects when present in appropriate or low concentrations, serving various physiological roles, including in cellular signaling pathways and immune responses. However, excessive production of free radicals can induce oxidative stress and impair cell function, leading to various inflammatory, autoimmune, and degenerative diseases (Di Domenico et al., 2014; Habib and Ibrahim, 2011). Consequently, organisms require endogenous and exogenous antioxidants to reduce oxidative stress (Liu et al., 2017). Synthetic nutritional supplements, classified as artificial exogenous antioxidants, are extensively used in the health functional food industry. However, rising living standards and increasing interest in individual healthcare have sparked a general desire to replace synthetic supplements with natural alternatives (Brown, 2016). Several studies indicate that plant-derived phenolic metabolites enhance their antioxidant ability (Kosseva and Webb, 2013; Lee et al., 2021b; Liu et al., 2021). Plant phenols are used for various purposes, primarily as reducing agents to counteract free radicals, and are present in all plant parts, including leaves, fruits, nuts, roots, bark, and seeds (Guleria et al., 2021; Nam et al., 2017).

The genus *Populus*, which includes poplars and belongs to the Salicaceae family, is predominantly distributed in temperate and subtropical regions. *Populus* spp. are deciduous trees that thrive in dry areas and propagate well from their roots owing to their shallow-rooted characteristics (Choi et al., 2020). These trees are primarily used for fast-growing plantation forestry, particularly in northern regions. They are a crucial fiber resource for the global pulp and paper industry owing to their high growth rates and favorable pulp properties (Greenwood Resources, 2014; Lu et al., 2006). Additionally, they possess significant medicinal value, containing high concentrations of salicylate-like phenolic glycosides and flavonoids. These salicylate-like phenolic glycosides are essential secondary metabolites in the genus *Populus* trees and other species within the Salicaceae family (Abreu et al., 2011). Salicylate compounds function as chemotaxonomic markers and chemical defenses against biotic stressors (Gondor et al., 2022; Khan et al., 2015). After ingestion, salicin is hydrolyzed and oxidized into salicylic acid, which acts similarly to aspirin (Mahdi et al., 2006). Additionally, various flavonoids, including (+)-catechin, eriodictyol, quercetin, kaempferol, pinobanksin, galangin, apigenin, luteolin, and myricetin are isolated from *Populus* spp. (Yang et al., 2023; Morreel et al., 2006; Tsai et al., 2006). Flavonoids are representative plant secondary metabolites with strong antioxidant properties (Zeng et al., 2013). Therefore, several *Populus* spp. have been used in traditional medicine since ancient times (Anđelković et al., 2017). *Populus nigra* and *P. tremula var. davidiana* exhibit pharmacological properties, including antioxidant, anti-inflammatory, and hepatoprotective effects (Debbache-Benaida et al., 2013; Zhang et al., 2006). Furthermore, *Populus balsamifera* shows pharmacological potential in treating obesity and diabetes and exhibits antibacterial properties (Harbilas et al., 2012; Simard et al., 2014). Currently, pharmacological companies extensively explore the pharmacological benefits of *Populus* spp.

Among *Populus* species, *P. × tomentiglandulosa* T.B.Lee (PT), known as “Silver aspen” was first reported as a natural hybrid between *P. alba* L. and *P. tremula* var. *glandulosa* Uyeki. Currently, this taxon is recognized as a Korean endemic plant (Chung et al., 2017). Although an artificial hybrid between the two species was made by Dr. S. G. Hyun, since a taxonomic treatment of this natural hybrid was already done (Son, 2009), we will be used this name, *P.* × *tomentiglandulosa* in this paper. Owing to its rapid growth, PT has been artificially hybridized and extensively used for afforestation and as street trees in South Korea. Numerous studies have reported the phytochemical metabolites and pharmacological activities of PT, highlighting its significant neuroprotective properties (Choi et al., 2020; Kwon and Koh, 2020; Lee et al., 2019a; Lee et al., 2019b; Park et al., 2017). Nevertheless, the isolation and analysis of phytochemical metabolites in PT and related species remain underexplored.

Therefore, this study aims to investigate various phytochemical constituents of PT leaves and stems using column chromatography, elucidate their structures through spectral analyses, and compare these metabolites with those of their parent species. In addition, we investigated the chemical profiling of PT and four fractions by assessing their radical scavenging activities. Additionally, anti-inflammatory properties of PT extracts and four fractions were demonstrated using lipopolysaccharide-induced Raw 264.7 macrophages.

## 2 Materials and methods

### 2.1 Plant materials

Dried PT leaves used for column chromatography were provided by the National Institute of Forest Science, Korea, in February 2020. The stems and leaves of PT, *P. alba*, and *P. tremula* var. *glandulosa* used for High-Performance Liquid Chromatography (HPLC) analysis and bioactivity assays were supplied by the National Institute of Forest Science, Korea, in July 2021. The individuals used in the analysis were planted in an experimental nursery at the National Institute of Forest Science, Suwon, Korea, and the plant specimens used were deposited in the Herbarium (KH) of Korea National Arboretum (Voucher number: No. KH20-10), Pocheon, Korea.

### 2.2 Apparatuses and chemicals

^1^H-nuclear magnetic resonance (NMR) and ^13^C-NMR spectra, heteronuclear single quantum coherence spectroscopy (HSQC), and heteronuclear multiple bond correlation (HMBC) spectra were recorded using an AVANCE Ⅲ HD 500 NMR spectrometer (Bruker, Hanau, Germany). The spectrometer operated at 500 and 125 MHz for ^1^H-NMR and ^13^C-NMR, respectively, and was equipped with a 5 mm BBFO (plus) probehead. Tetramethylsilane served as the internal standard. Fast atom bombardment mass spectrometry (FAB-MS) spectra were recorded using an MS-700 mass spectrometer (JEOL, Japan). Silica gel (60–200 mesh, Zeochem, Switzerland) and Sephadex LH-20 (Sigma-Aldrich, St. Louis, MO, United States) were used as stationary phases for open-column chromatography. Chromatographic separations were monitored using thin-layer chromatography (TLC) with pre-coated Kiesel gel 60 F_254_ plates (silica gel, 0.25 mm layer thickness, Art. 5715, Merck Co., Darmstadt, Germany). Compounds on TLC plates were visualized by spraying them with 10% H_2_SO_4_ in methanol (MeOH), followed by heating at 100°C on a hot plate. Solvents, including chloroform (CHCl_3_), ethyl acetate (EtOAc), *n-*butanol (*n*-BuOH), and MeOH, were purchased from Samchun Pure Chemicals (Pyeongtaek, Republic of Korea). HPLC-grade solvents such as water (H_2_O), MeOH, and acetonitrile (ACN) were obtained from J.T. Baker (Phillipsburg, PA, United States). The compound 1,1-Diphenyl-2-picrylhydrazyl (DPPH) was purchased from Alfa Aesar (London, United Kingdom), while 2,2′-azino-bis(3-ethylbenzothiazoline-6-sulfonic acid (ABTS^+^) was purchased from Roche (Mannheim, Germany). Phenazine methosulfate (PMS), nicotinamide adenine dinucleotide (NADH) disodium salt, nitrotetrazolium blue chloride (NBT), and thiobarbituric acid (TBA) were purchased from Sigma-Aldrich. Trichloroacetic acid (TCA) was purchased from Samchun Pure Chemicals. Absorbance readings were measured using a GloMax microplate reader (Madison, WI, United States).

### 2.3 Extraction, fractionation, and isolation procedures

The shade-dried, ground PT (956.4 g) was extracted with ethanol (EtOH, 8 L) under reflux at 83°C–84°C for 3 h, repeated five times. After extraction, the solvent was filtered through filter paper. It was then concentrated using a rotary evaporator (Eyela, Tokyo, Japan) at 55°C, yielding EtOH extracts (232.2 g). Subsequently, the extract was suspended with distilled water and sequentially partitioned with *n*-hexane, CHCl_3_, EtOAc, and H_2_O-saturated *n*-BuOH. The weights of the fractions obtained were as follows: *n*-hexane (25.1 g), CHCl_3_ (14.5 g), EtOAc (16.7 g), and *n*-BuOH (18.9 g). The EtOAc fraction (15 g) was subjected to silica gel column chromatography with a gradient of CHCl_3_–MeOH (from 100% CHCl_3_ to 100% MeOH), yielding nine subfractions. After TLC examination, fractions 4, 5, 6, and 7 from the first column were combined and loaded onto the Sephadex LH-20 column and gradually eluted with a gradient of H_2_O–MeOH (from 5:1 to 1:5) to obtain 12 subfractions. Subfractions 5 and 7 yielded compounds **3** and **2**, respectively. The *n*-BuOH fraction (18 g) was subjected to silica gel column chromatography using a gradient of CHCl_3_–MeOH (from 100% CHCl_3_ to 100% MeOH), resulting in six subfractions based on TLC experiments. Compounds **1** and **9** were obtained from subfractions 1 and 2, respectively, through MeOH recrystallization. The remaining subfractions were further subjected to column chromatography using Sephadex LH-20. All subfractions were loaded onto the Sephadex LH-20 column and eluted with a gradient of H_2_O–MeOH (from 5:1 to 1:5) to purify the compounds. Subfractions 3 and 4 yielded compounds **7** and **8**, respectively, while subfraction 5 yielded compound **6**, and subfraction 6 yielded compounds **4** and **5**.

Compound **1**: White powder; FAB-MS *m/z*: 309 [M + Na]^+^; ^1^H-NMR (500 MHz, DMSO-*d*_6_) δ: 4.76 (1H, d, *J* = 7.5 Hz, Glc-1), 5.08 (1H, d, *J* = 4.5 Hz, H_a_-7), 5.35 (1H, d, *J* = 5.0 Hz, H_b_-7), 7.00 (1H, dt, *J* = 1.0, 7.5 Hz, H-4), 7.09 (1H, dd, *J* = 1.0, 8.5 Hz, H-6), 7.19 (1H, dt, *J* = 1.5, 7.9 Hz, H-5), 7.35 (1H, dd, *J* = 1.5, 7.5 Hz, H-3). ^13^C-NMR (125 MHz, DMSO-*d*_6_) δ: 154.7 (C-1), 131.5 (C-2), 127.2 (C-3), 121.8 (C-4), 127.7 (C-5), 114.8 (C-6), 58.28 (C-7), 101.4 (Glc C-1), 73.4 (Glc C-2), 76.5 (Glc C-3), 69.8 (Glc C-4), 77.1 (Glc C-5), 60.8 (Glc C-6).

Compound **2**: White powder; FAB-MS *m/z*: 429 [M + Na]^+^; ^1^H-NMR (500 MHz, DMSO-*d*_6_) δ: 4.65 (1H, d, *J* = 7.5 Hz, Glc-1), 5.39 (2H, s, H-7), 6.68 (1H, dd, *J* = 3.0, 9.0 Hz, H-4), 6.80 (1H, d, *J* = 3.0, H-6), 7.04 (1H, d, *J* = 9.0 Hz, H-3), 7.52 (2H, br t, *J* = 7.8 Hz, H-3′, 5′), 7.68 (1H, br t, *J* = 7.5 Hz, H-4′), 8.02 (2H, dd, *J* = 1.3, 8.3 Hz, H-2′, 6′). ^13^C-NMR (125 MHz, DMSO-*d*_6_) δ: 126.7 (C-1), 147.9 (C-2), 117.8 (C-3), 114.6 (C-4), 152.4 (C-5), 115.3 (C-6), 61.7 (C-7), 129.8 (C-1′), 129.3 (C-2′), 128.9 (C-3′), 133.5 (C-4′), 128.9 (C-5′), 129.3 (C-6′), 165.7 (C-7′), 102.7 (Glc C-1), 73.5 (Glc C-2), 77.1 (Glc C-3), 69.9 (Glc C-4), 76.6 (Glc C-5), 60.9 (Glc C-6).

Compound **3**: White powder; FAB-MS *m/z*: 413 [M + Na]^+^; ^1^H-NMR (500 MHz, DMSO-*d*_6_) δ: 5.24 (1H, d, *J* = 8.0 Hz, Glc-1), 5.34 (1H, d, *J* = 5.0 Hz, H_a_-7), 5.49 (1H, d, *J* = 5.5 Hz, H_b_-7), 6.98 (1H, dt, *J* = 1.0, 7.4 Hz, H-4), 7.08 (1H, dd, *J* = 1.0, 8.5, H-6), 7.17 (1H, dt, *J* = 1.5, 7.8 Hz, H-5), 7.31 (1H, dd, *J* = 2.0, 7.5 Hz, H-3), 7.52 (2H, br t, *J* = 7.8 Hz, H-3′, 4′), 7.65 (1H, br t, *J* = 7.3 Hz, H-4′), 7.99 (2H, dd, *J* = 1.5, 8.5 Hz, H-2′, 6′). ^13^C-NMR (125 MHz, DMSO-*d*_6_) δ: 153.4 (C-1), 131.1 (C-2), 127.3 (C-3), 121.9 (C-4), 126.3 (C-5), 113.9 (C-6), 57.2 (C-7), 129.8 (C-1′), 129.3 (C-2′), 128.7 (C-3′), 133.3 (C-4′), 128.7 (C-5′), 129.3 (C-6′), 165.0 (C-7′), 98.2 (Glc C-1), 73.8 (Glc C-2), 77.2 (Glc C-3), 69.9 (Glc C-4), 74.2 (Glc C-5), 60.5 (Glc C-6).

Compound **4**: Yellow powder; FAB-MS *m/z*: 463 [M + H]^+^; ^1^H-NMR (500 MHz, DMSO-*d*_6_) 3.89 (3H, s, OCH_3_), 5.06 (1H, d, *J* = 7.0 Hz, Glc-1), 6.45 (1H, d, *J* = 2.0 Hz, H-6), 6.87 (1H, d, *J* = 2.5 Hz, H-8), 6.94 (1H, d, *J* = 8.0 Hz, H-5′), 6.99 (1H, s, H-3), 7.60 (1H, s, H-2′), 7.59 (1H, dd, *J* = 2.0, 9.0 Hz, H-6′), 12.97 (1H, s, 5-OH). ^13^C-NMR (125 MHz, DMSO-*d*_6_) δ: 164.2 (C-2), 103.1 (C-3), 182.1 (C-4), 161.1 (C-5), 99.5 (C-6), 163.0 (C-7), 95.0 (C-8), 156.9 (C-9), 105.3 (C-10), 121.2 (C-1′), 110.3 (C-2′), 148.1 (C-3′), 151.2 (C-4′), 115.8 (C-5′), 120.6 (C-6′), 99.9 (Glc C-1), 73.1 (Glc C-2), 76.4 (Glc C-3), 69.6 (Glc C-4), 77.2 (Glc C-5), 60.6 (Glc C-6), 56.0 (3′-OCH_3_).

Compound **5**: Yellow powder; FAB-MS *m/z*: 449 [M + H]^+^; ^1^H-NMR (500 MHz, DMSO-*d*_6_) δ: 5.07 (1H, d, *J* = 7.5 Hz, Glc-1), 6.43 (1H, s, H-6), 6.71 (1H, s, H-3), 6.78 (1H, s, H-8), 6.85 (1H, d, *J* = 8.5 Hz, H-5′), 7.40 (1H, d, *J* = 1.5 Hz, H-2′), 7.43 (1H, dd, *J* = 2.0, 8.5 Hz, H-6′), 13.05 (1H, s, 5-OH). ^13^C-NMR (125 MHz, DMSO-*d*_6_) δ: 164.6 (C-2), 102.9 (C-3), 181.8 (C-4), 161.1 (C-5), 99.5 (C-6), 162.9 (C-7), 94.7 (C-8), 156.9 (C-9), 105.3 (C-10), 120.7 (C-1′), 113.2 (C-2′), 146.0 (C-3′), 150.8 (C-4′), 116.0 (C-5′), 119.3 (C-6′), 99.9 (Glc C-1), 73.1 (Glc C-2), 77.2 (Glc C-3), 69.5 (Glc C-4), 76.4 (Glc C-5), 60.6 (Glc C-6).

Compound **6**: Yellow powder; FAB-MS *m/z*: 611 [M + H]^+^; ^1^H-NMR (500 MHz, DMSO-*d*_6_) δ: 0.99 (3H, d, *J* = 6.0 Hz, Rha-CH_3_), 4.38 (1H, d, *J* = 1.0 Hz, Rha-1), 5.34 (1H, d, *J* = 7.5 Hz, Glc-1), 6.19 (1H, d, *J* = 2.0 Hz, H-6), 6.38 (1H, d, *J* = 2.0 Hz, H-8), 6.83 (1H, d, *J* = 8.5 Hz, H-5′), 7.52 (1H, d, *J* = 2.0 Hz, H-2′), 7.53 (1H, dd, *J* = 2.3, 10.8 Hz, H-6′), 12.59 (1H, s, 5-OH). ^13^C-NMR (125 MHz, DMSO-*d*_6_) δ: 156.5 (C-2), 133.3 (C-3), 177.4 (C-4), 161.2 (C-5), 98.7 (C-6), 164.2 (C-7), 93.6 (C-8), 156.6 (C-9), 103.9 (C-10), 121.6 (C-1′), 115.2 (C-2′), 144.8 (C-3′), 148.5 (C-4′), 116.3 (C-5′), 121.2 (C-6′), 101.2 (Glc C-1), 74.1 (Glc C-2), 76.5 (Glc C-3), 70.0 (Glc C-4), 75.9 (Glc C-5), 67.0 (Glc C-6), 100.8 (Rha C-1), 70.4 (Rha C-2), 70.6 (Rha C-3), 71.9 (Rha C-4), 68.2 (Rha C-5), 17.8 (Rha C-6).

Compound **7**: Yellow powder; FAB-MS *m/z*: 625 [M + H]^+^; ^1^H-NMR (500 MHz, DMSO-*d*_6_) δ: 0.97 (3H, d, *J* = 6.0 Hz, Rha-CH_3_), 3.83 (3H, s, OCH_3_), 4.41 (1H, d, *J* = 1.0 Hz, Rha-1), 5.43 (1H, d, *J* = 7.5 Hz, Glc-1), 6.20 (1H, d, *J* = 2.0 Hz, H-6), 6.43 (1H, d, *J* = 2.0 Hz, H-8), 6.90 (1H, d, *J* = 8.0 Hz, H-5′), 7.51 (1H, dd, *J* = 2.0, 8.5 Hz, H-6′), 7.85 (1H, d, *J* = 2.5 Hz, H-2′), 12.57 (1H, s, 5-OH). ^13^C-NMR (125 MHz, DMSO-*d*_6_) δ: 156.5 (C-2), 133.0 (C-3), 177.3 (C-4), 161.2 (C-5), 98.7 (C-6), 164.2 (C-7), 93.8 (C-8), 156.5 (C-9), 104.0 (C-10), 121.0 (C-1′), 113.3 (C-2′), 146.9 (C-3′), 149.4 (C-4′), 115.2 (C-5′), 122.3 (C-6′), 101.2 (Glc C-1), 74.3 (Glc C-2), 75.9 (Glc C-3), 70.1 (Glc C-4), 76.4 (Glc C-5), 66.8 (Glc C-6), 100.9 (Rha C-1), 70.3 (Rha C-2), 70.6 (Rha C-3), 71.8 (Rha C-4), 68.3 (Rha C-5), 17.7 (Rha C-6), 55.6 (3′-OCH_3_).

Compound **8**: Yellow powder; FAB-MS *m/z*: 519 [M + H]^+^; ^1^H-NMR (500 MHz, DMSO-*d*_6_) δ: 0.83 (3H, t, *J* = 7.5 Hz, H-4‴), 1.32 (2H, m, H-3‴), 1.55 (2H, m, H-2‴), 4.03–4.09 (2H, m, H-1‴), 4.12 (1H, d, *J* = 9.5 Hz, Glc-5), 5.32 (1H, d, *J* = 7.0 Hz, Glc-1), 6.46 (1H, d, *J* = 2.0 Hz, H-6), 6.75 (1H, s, H-3), 6.81 (1H, d, *J* = 2.0 Hz, H-8), 6.89 (1H, d, *J* = 8.0 Hz, H-5′), 7.41 (1H, d, *J* = 2.0 Hz, H-2′), 7.44 (1H, dd, *J* = 2.3, 8.3 Hz, H-6′), 13.01 (1H, s, 5-OH). ^13^C-NMR (125 MHz, DMSO-*d*_6_) δ: 168.7 (C-2), 103.0 (C-3), 181.8 (C-4), 162.4 (C-5), 99.1 (C-6), 164.5 (C-7), 94.5 (C-8), 161.1 (C-9), 105.4 (C-10), 121.1 (C-1′), 113.4 (C-2′), 145.9 (C-3′), 150.3 (C-4′), 115.9 (C-5′), 119.1 (C-6′), 99.3 (Glc C-1), 72.7 (Glc C-2), 75.4 (Glc C-3), 71.2 (Glc C-4), 75.1 (Glc C-5), 169.2 (Glc C-6), 64.3 (C-1‴), 30.0 (C-2‴), 18.4 (C-3‴), 13.5 (C-4‴).

Compound **9**: Yellow powder; FAB-MS *m/z*: 533 [M + H]^+^; ^1^H-NMR (500 MHz, DMSO-*d*_6_) δ: 0.83 (3H, t, *J* = 7.5 Hz, H-4‴), δ 1.31 (2H, m, H-3‴), δ 1.56 (2H, m, H-2‴), 3.90 (3H, s, OCH_3_), 4.03–4.13 (2H, m, H-1‴), 4.18 (1H, d, *J* = 9.5 Hz, Glc-5), 5.31 (1H, d, *J* = 7.0 Hz, Glc-1), 6.48 (1H, d, *J* = 2.5 Hz, H-6), 6.87 (1H, d, *J* = 2.0 Hz, H-8), 6.95 (1H, d, *J* = 9.0 Hz, H-5′), 7.00 (1H, s, H-3), 7.59 (1H, dd, *J* = 2.0, 8.8 Hz, H-6′), 7.60 (1H, d, *J* = 2.0 Hz, H-2′), 12.99 (1H, s, 5-OH). ^13^C-NMR (125 MHz, DMSO-*d*_6_) δ: 164.2 (C-2), 103.5 (C-3), 182.1 (C-4), 161.2 (C-5), 99.3 (C-6), 162.4 (C-7), 94.9 (C-8), 156.9 (C-9), 105.5 (C-10), 121.3 (C-1′), 110.4 (C-2′), 148.1 (C-3′), 151.0 (C-4′), 115.8 (C-5′), 120.5 (C-6′), 99.4 (Glc C-1), 72.8 (Glc C-2), 75.5 (Glc C-3), 71.2 (Glc C-4), 75.2 (Glc C-5), 168.7 (Glc C-6), 64.4 (C-1‴), 30.0 (C-2‴), 18.5 (C-3‴), 13.5 (C-4‴), 56.0 (3′-OCH_3_).

### 2.4 Sample preparation for HPLC and HPLC conditions

To quantify compounds **1–9** in each *Populus* spp. extract, 45 mg of each extract was dissolved in 3 mL of MeOH to prepare a stock solution. The resulting solutions were filtered through a Whatman 0.45-μm polyvinylidene difluoride (PVDF) syringe filter (Cat No. 6779, Whatman plc, Maidstone, United Kingdom) before HPLC analysis. A reverse-phase HPLC system with a YMC-Pack Pro C18 column (25 cm × 4.6 mm, 5 μm) was used for simultaneous analysis of the nine compounds. The injection volume was 10 μL, and detection was monitored at 252 nm. The column oven temperature was maintained at 30°C, while the flow rate was set at 1.0 mL/min. A gradient elution system was used for the analysis. The mobile phase consisted of 0.25% acetic acid in water (A) and ACN (B). Elution gradient was conducted as follows: 90% A at 0 min and maintained until 10 min; 80% A from 10 to 20 min; 70% A from 20 to 30 min; 50% A from 30 to 45 min; 0% A from 45 to 50 min and maintained until 55 min; and 90% A from 55 to 60 min, maintained until 70 min.

### 2.5 Calibration curves

Standard stock solutions (0.5–1 mg/mL) of compounds **1**–**9** isolated from PT were prepared in MeOH and repeatedly mixed with the same solvent. The purity of the isolated compounds was determined by HPLC analysis, with all compounds exhibiting a purity of greater than 94%. Calibration curves were plotted by correlating the concentrations of the standard solutions with their respective peak areas. The linearity of the calibration curves was determined using the correlation coefficient (*r* ^2^). Subsequently, the concentrations of compounds **1**–**9** in the samples were calculated from the calibration curves. The calibration functions were derived from the peak area (*Y*) and concentration (*X*, μg/mL), with results presented as mean ± standard deviation (*n* = 3) (Table 1).

**TABLE 1:** Calibration curves of compounds **1**–**9**.

Compound	t_R_	Calibration equation	Correlation factor, *r* ^2^
Salicin (**1**)	8.4	Y = 732.57X **+** 12,700	0.9997
Salireposide (**2**)	31.2	Y = 2176.6X **+** 44,278	0.9996
Populin (**3**)	35.0	Y = 2247.3X **+** 6126	0.9997
Thermopsoside (**4**)	30.6	Y = 186,61X **+** 10,228	0.9996
Cynaroside (**5**)	27.7	Y = 225,27X **+** 20,287	0.9999
Rutin (**6**)	26.3	Y = 155,00X **+** 113,515	0.9994
Narcissin (**7**)	29.6	Y = 153,78X − 16,025	0.9996
Luteolin 7-*O*-*β*-d-glucuronide butyl ester (**8**)	41.0	Y = 6757X **+** 1,699.2	0.9999
Chrysoeriol 7-*O*-*β*-d-glucuronide butyl ester (**9**)	44.3	Y = 104,37X **+** 16,938	0.9994

t_R_ = retention time. Y = peak area, X = concentration of the standard (μg/mL). *r* ^2^ = correlation coefficient for data points on the calibration curve.

### 2.6 DPPH radical scavenging activity

DPPH radical scavenging activity was measured as described by Kwon et al. (2020). The extract and four fractions, including the active compounds from PT, were dissolved in EtOH. Subsequently, the samples were mixed with a 60-μM DPPH solution in 96-well plates and incubated in the dark at room temperature. After 30 min, absorbance at 540 nm was measured using a microplate reader. DPPH radical scavenging activity was calculated using the formula: DPPH radical scavenging activity (%) = [(Abs_c_ − Abs_s_)/Abs_c_] × 100, where Abs_c_ and Abs_s_ represent the absorbance of the control and sample, respectively.

### 2.7 ABTS^+^ radical scavenging activity

ABTS^**+**^ radical scavenging activity was measured as previously described by Thaipong et al. (2006). The ABTS^**+**^ solution was prepared by reacting 2,2′-azinobis-(3-ethylbenzothiazolin-6-sulfonic acid) (ABTS, 7.4 mM) with potassium persulfate (2.6 mM) for 12–16 h in the dark until the oxidation-reduction reaction occurred and ABTS^**+**^ was formed. The working ABTS solution was prepared by diluting the stock solution with purified water until the absorbance reached 1.0 at 600 nm. Subsequently, the fraction samples were mixed with the working ABTS^**+**^ solution in 96-well plates and incubated in the dark at room temperature. After 30 min, the absorbance at 600 nm was measured using a microplate reader with l-ascorbic acid serving as the positive control. ABTS^**+**^ radical scavenging activity was calculated using the formula: ABTS^**+**^ radical scavenging activity (%) = [(Abs_c_ − Abs_s_)/Abs_c_] × 100, where Abs_c_ and Abs_s_ represent the absorbance of the control and sample, respectively.

### 2.8 ^•^OH radical scavenging activity

^•^OH radical scavenging activity was measured using the method described by Kwon et al. (2020). The fractions of PT were evaporated to dryness to remove the solvent (Li, 2013). Subsequently, four fractions of PT were dissolved in phosphate-buffered saline and mixed with 10 mM FeSO_4_•7H_2_O-EDTA, 10 mM 2-deoxyribose, and 10 mM H_2_O_2_. The mixtures were incubated at 37°C in the dark for 4 h. After this, 1% TBA and 2.8% TCA solutions were added, and the mixtures were heated to 100°C for 20 min. After cooling, the absorbance was measured at 490 nm using a microplate reader. l-ascorbic acid was used as the positive control. ^•^OH radical scavenging activity was calculated using the formula: ^•^OH radical scavenging activity (%) = [(Abs_c_ − Abs_s_)/Abs_c_] × 100, where Abs_c_ demotes the absorbance of the control, and Abs_s_ denotes the absorbance of the sample.

### 2.9 O_2_^−^ radical scavenging activity

O_2_^−^ radical scavenging activity was assessed following the method described by Kwon (2020). Four fractions of PT diluted in H_2_O were mixed with 0.1 M Tris-HCl (pH 7.4), 200 μM PMS, 1 mM NBT, and 1 mM NADH and incubated at room temperature in the dark. After 10 min, absorbance was measured at 560 nm using a microplate reader. l-ascorbic acid was used as the positive control. O_2_^−^ radical scavenging activity was calculated using the formula: O_2_^−^ radical scavenging activity (%) = [(Abs_c_ − Abs_s_)/Abs_c_] × 100, where Abs_c_ denotes the absorbance of the control, and Abs_s_ denotes the absorbance of the sample.

### 2.10 Cell viability assay using MTT assay

The murine macrophage cell line, RAW 264.7, was purchased from the Korean Cell Line Bank (Seoul, Republic of Korea). The cells were cultured in Dulbecco’s Modified Eagle Medium (DMEM; Gibco, Paisley, United Kindom) supplemented with 10% fetal bovine serum (FBS; Gibco) and 1% penicillin/streptomycin (Gibco) and incubated at 37°C in a 5% CO_2_ atmosphere. The 3-(4,5-dimethylthiazol-2-yl)-2,5 diphenyl tetrazolium bromide (MTT) assay was used to assess cellular metabolic activity as an indicator of cell viability, proliferation, and cytotoxicity. RAW 264.7 cells (1 × 10^4^ cells/well) were seeded in 96-well plates and incubated for 24 h. Subsequently, the cells were exposed to various concentrations (0.01–1,000 μg/mL) of samples for an additional 24 h. MTT solution (0.5 mg/mL, 100 μL) was added to each well, and the cells were further incubated for ∼3 h at 37°C. The resulting formazan crystals were dissolved in dimethyl sulfoxide (DMSO; 100 μL), and the absorbance was measured at 570 nm using a microplate reader (Molecular Devices Filter Max F5; San Francisco, CA, United States).

### 2.11 Evaluation of anti-inflammatory effects using western blot analysis

The Raw 264.7 cells were seeded into 6-well plates at a density of 5 × 10^5^ cells/well. After 18 h, the cells were exposed to PT fractions at various concentrations (0.1–100 μg/mL) at 1 h before stimulation with lipopolysaccharides (0127:B8; 1 μg/mL; Sigma-Aldrich). After 5 h of lipopolysaccharide treatment, the cells were collected for Western blot analysis. The Western blot assay was conducted following previously described methods (Choi et al., 2021a; Choi et al., 2021b; Jang et al., 2019; Lee et al., 2021a). Briefly, the Raw 264.7 cells were homogenized in a cell lysis buffer containing 10 mM Tris-Cl (pH 7.4), 0.5 mM ethylenediaminetetraacetic acid, 0.25 M sucrose, and a protease inhibitor mixture using a bullet blender tissue homogenizer (Next Advance, Inc., Troy, NY, United States). The Western blot assay, protein transfer to PVDF membranes, and membrane blocking were conducted following previously described methods (Choi et al., 2021a; Choi et al., 2021b; Jang et al., 2019; Lee et al., 2021a). The PVDF membranes were incubated with primary antibodies, including rat anti-interleukin (IL)-6 (1:500, Cell Signaling Technology, Danvers, MA, United States), rat anti-inducible nitric oxide (1:500, Cell Signaling Technology), and rabbit anti-GAPDH (Cell Signaling Technology), followed by incubation with horseradish peroxidase–conjugated secondary antibody (1:500, Vector Laboratories, Newark, CA, United States). Blot signals were detected using an enhanced chemiluminescence kit (Merck KGaA, Darmstadt, Germany). Each Western blot analysis was conducted in triplicate and independently repeated three times, yielding consistent results. Protein band density was measured using ImageJ software.

### 2.12 Statistical analysis

All results were presented as mean ± SD. Statistical significance (*p* < 0.05) was determined using analysis of variance (ANOVA) followed by Duncan’s multiple range tests or Tukey’s *post hoc* test using the Statistical Package for the Social Sciences (SPSS, Chicago, IL, Unites States).

## 3 Results and discussion

### 3.1 Identification of phytochemical compounds in PT extract

**FIGURE 1:**
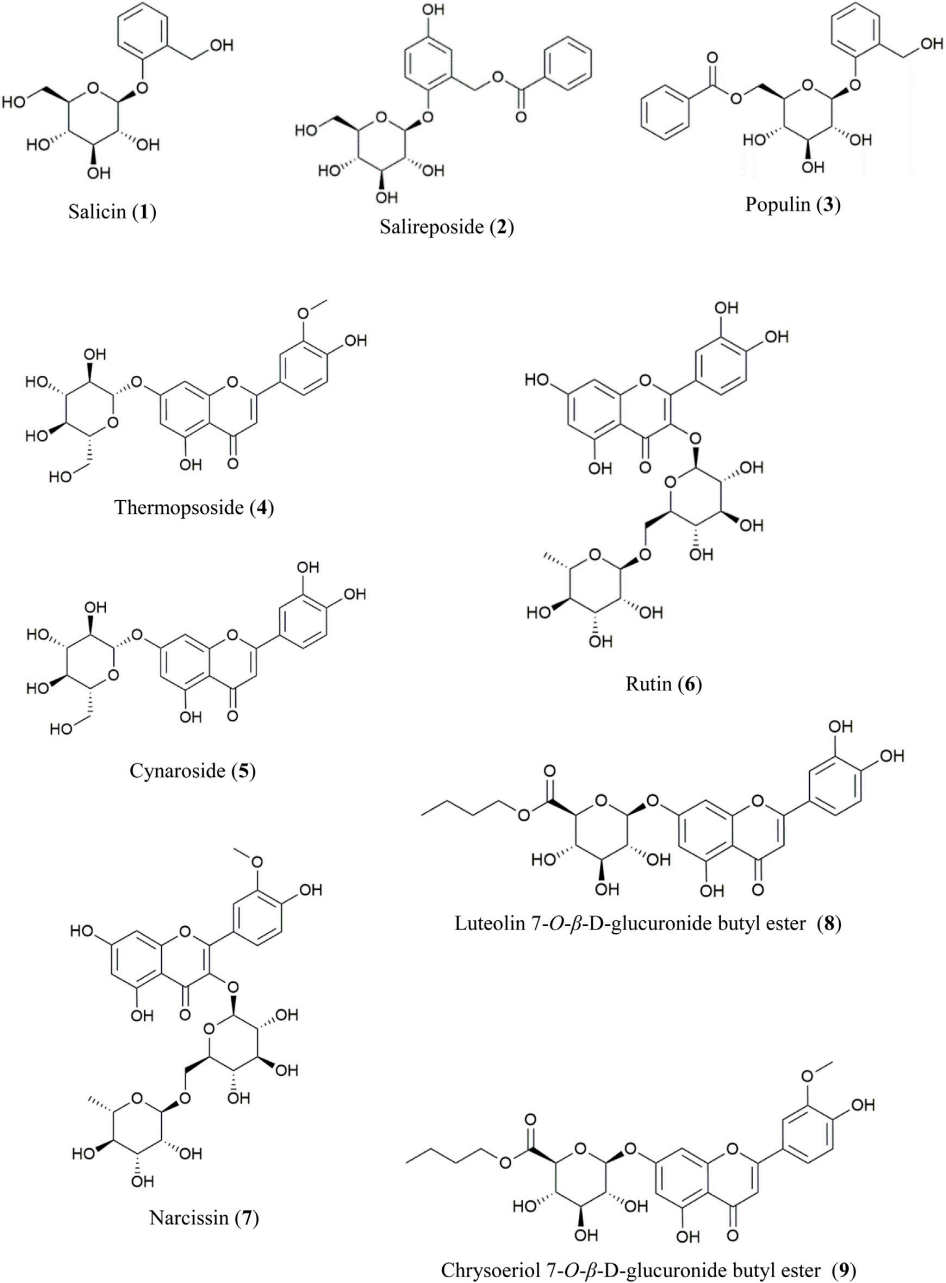
Chemical structures of compounds **1**–**9** from PT.

The EtOAc- and *n*-BuOH-soluble fractions of PT were chromatographically separated, leading to the isolation of compounds **1**–**9** through repeated column chromatography. Their structures were elucidated using spectroscopic analysis and comparison with literature values (Figure 1).

From the EtOAc-soluble fraction, three salicylate-like phenolic compounds were isolated. Compounds **1**–**3** were obtained as white powders. Compound **1** exhibited a molecular ion peak at *m/z* 309 [M + Na]^+^ in the FAB-MS, corresponding to the molecular formula C_13_H_18_O_7_. Compound **2** displayed a molecular ion peak at *m/z* 429 [M + Na]^+^ in the FAB-MS, corresponding to the molecular formula C_20_H_22_O_9_, while compound 3 showed a molecular ion peak at *m/z* 413 [M + Na]^+^ in the FAB-MS, corresponding to the molecular formula C_20_H_22_O_8_. The NMR spectra of compounds **1**–**3** exhibited characteristic signals with salicylate-like phenolic glycosides. The ^1^H NMR spectra revealed characteristic signals of a salicyl alcohol unit ranging from δ 6.68 to δ 7.35 and a glucosyl moiety appearing between δ 3.30 and δ 5.24. Only compounds 2 and 3 exhibited benzoyl protons between δ 7.52 and δ 8.02 in their 1H NMR spectra. Compound 2 showed one less hydrogen integral than compound 3 in the downfield region of the 1H NMR spectrum, attributable to the presence of gentisyl alcohol (5-OH) in compound 2 and salicyl alcohol (4-H) in compound **3**. Based on the ^13^C-NMR spectra, compounds 2 and 3 with a benzoyl group displayed seven carbon peaks ranging from δ 128.7 to δ 165.7. The peaks at δ 165.0 and δ 165.7 corresponded to the ester linkage between benzoic acid and a sugar unit. Additionally, in the spectra of compounds 1–3, peaks corresponding to the glucosyl moiety were observed between δ 60.5 and δ 77.2, with the anomeric carbon appearing at δ 101.4, δ 102.7, and δ 98.2, respectively. Among the carbons associated with salicyl alcohol, C-7 was observed in the upfield region around δ 57.2–δ 61.7, while the remaining carbons were detected between δ 113.9and δ 154.7. Since compound **2** has a gentisyl alcohol structure, C-5 was downfield shifted by a hydroxyl group, appearing at δ 152.4. Consequently, the structures of compounds **1**, **2**, and **3** were identified as salicin, salireposide, and populin, respectively. All acquired spectroscopic data were analyzed and compared with literature values (Ahmad et al., 2003; Kumari et al., 2016; Xu et al., 2018).

Various flavonoid glycosides were isolated from the *n*-BuOH-soluble fraction. In the FAB-MS, all isolated compounds exhibited molecular ion peaks consistent with their corresponding molecular formulas, as described in the Materials and Methods. Typical flavonoid signals were observed in the ^1^H–NMR spectra of compounds **4**–**9**. The presence of singlet signals at δ 12.57–δ 13.01 indicated the 5-OH group of the A-ring in the structure. A pair of *meta*-coupled aromatic proton signals (d, *J* = 2.0–2.5 Hz) localized at δ 6.19–δ 6.94 corresponded to the H-6 and H-8 protons of the A-ring. Additionally, a *meta*-coupled doublet corresponded to H-2′ at δ 7.50–δ 7.85 (1H, d, *J* = 2–2.5 Hz), and an *ortho*-*meta*-coupled doublet-doublet corresponded to H-6′ at δ 7.43–δ 7.60 (1H, dd, *J* = 2–2.3, 8–8.8 Hz). The *ortho*-coupled doublet H-5′ at δ 6.83–δ 6.95 (1H, d, *J* = 8–8.5 Hz) exhibited ABX splitting signals indicative of the B-ring structure in flavonoids. In compounds **4** and **5**, the anomeric proton signal of the glucosyl moiety was detected at δ 5.06 (1H, d, *J* = 7.0 Hz, Glc-1) and δ 5.07 (1H, d, *J* = 7.5 Hz, Glc-1), respectively. In each ^13^C-NMR spectrum, a signal at δ 99.9 corresponded to the anomeric carbon of d-glucose. Additionally, in the ^1^H-NMR spectra of compounds **4** and **7**, the methoxy signals were observed at δ 3.89 and δ 3.83, respectively. The downfield shift of the H-2′ signal relative to the H-6′ signal suggests the presence of a 3′-methoxy-4′-hydroxy moiety in the B-ring. In compounds **6** and **7**, each of the two anomeric protons of sugars were observed at δ 5.34 (1H, d, *J* = 7.5 Hz, Glc-1) and δ 4.38 (1H, d, *J* = 1.0 Hz, Rha-1) and δ 5.43 (1H, d, *J* = 7.5 Hz, Glc-1), δ 4.41 (1H, d, *J* = 1.0 Hz, Rha-1), respectively. Additionally, the rhamnosyl CH_3_ signals were observed in the upfield region at δ 0.99 and δ 0.97 (3H, d, *J* = 6.0 Hz) for compounds **6** and **7**, respectively. In the ^13^C-NMR spectra of compounds **6** and **7**, two peaks at δ 101.2, δ 100.8, and 101.2, δ 100.9, were assignable to the two anomeric carbons of d-glucose and l-rhamnose, respectively. The remaining sugar-related peaks of compound **7** were similar to those of compound **6**, suggesting the presence of the same sugar: rutinose. Based on these interpretations and direct comparisons with published literature, compounds **4**, **5**, **6**, and **7** were identified as thermopsoside (chrysoeriol 7-*O*-glucoside), cynaroside (luteolin 7-*O*-glucoside), rutin (quercetin-3-*O*-rutinoside), and narcissin (isorhamnetin 3-*O*-rutinoside), respectively (Akbari-Ahangar and Delnavazi, 2020; Ilhan et al., 2019; Shojaeifard et al., 2021).

Compound **8** was recrystallized from MeOH as a yellow powder. A molecular ion peak was measured at *m/z*: 519 [M + H]^+^ in the FAB-MS (positive mode), suggesting a molecular formula of C_25_H_26_O_12_. In the HSQC spectrum, correlations were observed between H-3 (δ 6.75) and C-3 (δ 103.0), H-6 (δ 6.46) and C-6 (δ 99.1), H-8 (δ 6.81) and C-8 (δ 94.5), H-2′ (δ 7.41) and C-2′ (δ 113.4), H-5′ (δ 6.89) and C-5′ (δ 115.9), and H-6′ (δ 7.44) and C-6′ (δ 119.1). These correlations indicate the presence of a 5,7,3′,4′-tetrasubstituted flavone (luteolin) in the structure. An anomeric proton signal at δ 5.32 (1H, d, *J* = 7.0 Hz, H-1″) showed an HSQC correlation with the corresponding anomeric carbon at δ 99.3 (C-1″). The sugar moiety was identified as glucuronic acid through additional characteristic signals of glucuronic acid [δ 4.12 (1H, d, *J* = 9.5 Hz, H-5″), δ 169.2 (C-6″)]. The *β*-configuration and d-configuration were deduced from the coupling constant of 7.0 Hz (Ashenhurst, 2022) and literature (Nzowa et al., 2010), respectively. Additionally, each proton signal appearing at δ 4.03–4.09 (2H, m, H-1‴), δ 1.55 (2H, m, H-2‴), δ 1.32 (2H, m, H-3‴), δ 0.83 (3H, t, *J* = 7.5 Hz, H-4‴) showed HSQC correlations with the carbon signals at δ 64.3 (C-1‴), δ 30.0 (C-2‴), δ 18.4 (C-3‴), and δ 13.5 (C-4‴), respectively. This corresponds to a butyl group composed of three methylene and one methyl group. The connectivity of the luteolin aglycone, glucuronic acid moiety, and butyl ester group was established through HMBC correlations. Correlations were specifically observed between the anomeric proton at δ 5.32 (H-1″) and carbon signal at δ 164.5 (C-7), indicating their spatial proximity. Additionally, correlations were observed between the oxymethylene protons of the butyl ester group at δ 4.03–4.09 (H-1‴) and the carbonyl carbon of the glucuronic acid moiety at δ 169.2 (C-6″), confirming their structural connectivity. Based on the data explained above and comparisons with spectral data from previously reported values (Shi-hui, 2012), the structure of compound 8 was identified as luteolin 7-*O*-*β*-d-glucuronide butyl ester.

Compound **9** was recrystallized from MeOH, resulting in a yellow powder. The FAB-MS (positive mode) showed a molecular ion peak at *m/z*: 533 [M + H]^+^, suggesting a molecular formula of C_25_H_28_O_13_. The ^1^H and ^13^C-NMR spectra closely resembled those of compound 8, with significant differences. In the ^1^H-NMR spectrum, a prominent singlet was observed at δ 3.90 (3H, s), indicative of a methoxy group, which was also observed in the ^13^C-NMR at δ 56.0. Furthermore, the HMBC correlation between the methoxy group at δ 3.90 (OCH_3_) and the carbon signal at δ 148.1 (C-3′) confirmed the presence of a 3′-methoxy in the flavonoid B ring. Additionally, the HMBC correlations showed the anomeric proton at δ 5.31 (H-1″) connected to the carbon signal at δ 162.4 (C-7) and the oxymethylene protons of the butyl ester group at δ 4.03–4.13 (H-1‴) connected to the carbonyl carbon of the glucuronic acid moiety at δ 168.7(C-6″), as observed in compound 8. By comparing these spectra with those of compound 8 and the previously mentioned data, the structure of compound **9** was determined to be chrysoeriol 7-*O*-*β*-d-glucuronide butyl ester. This structure was further validated by comparison with previously reported values (Wei et al., 2018).

Of the nine isolated compounds, populin (**3**), thermopsoside (**4**), cynaroside (**5**), rutin (**6**), narcissin (**7**), luteolin 7-O-glucuronide butyl ester (**8**), and chrysoeriol 7-*O*-glucuronide butyl ester (**9**) were identified from PT for the first time. Additionally, luteolin 7-*O*-*β*-d-glucuronide butyl ester (**8**) and chrysoeriol 7-*O*-*β*-d-glucuronide butyl ester (**9**) were identified from the *Populus* genus for the first time.

### 3.2 HPLC analysis comparing compounds 1-9 content in *Populus* species

**FIGURE 2:**
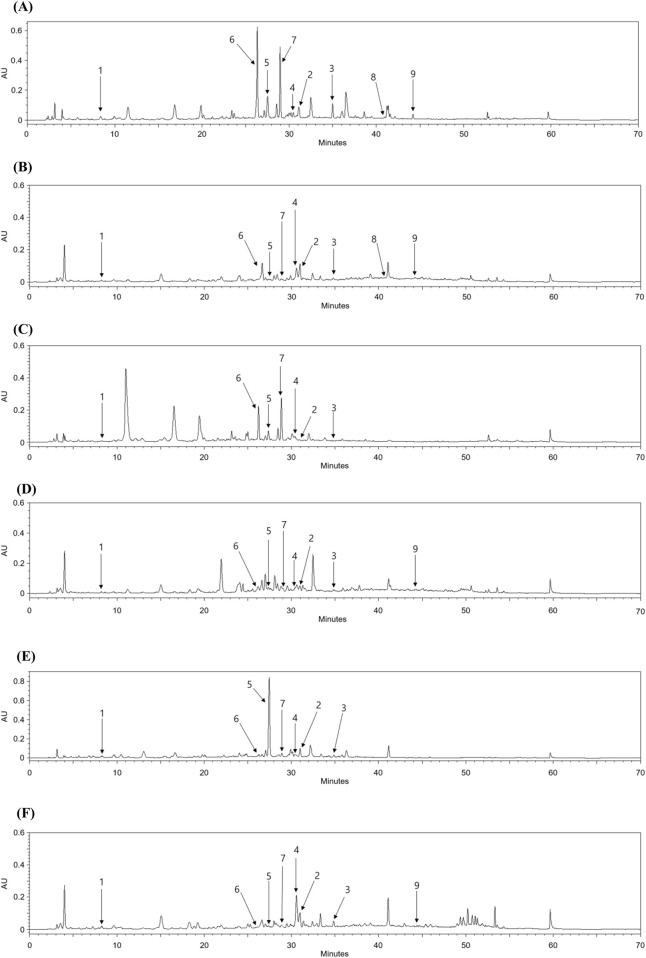
HPLC chromatograms of the leaves **(A)** and stems **(B)** of PT, leaves **(C)** and stems **(D)** of *P. alba*, and leaves **(E)** and stems **(F)** of *P. glandulosa*.

HPLC quantitative analysis was performed on PT leaves following chromatographic isolation. To compare plant parts and species, the leaves and stems of *P. alba* and *P. glandulosa*, along with PT, were analyzed (Table 2). Furthermore, an appropriate HPLC method for the simultaneous analysis of the nine isolated compounds was developed. A UV wavelength of 252 nm was employed for the simultaneous analysis of salicylate-like phenolic compounds from the EtOAc fraction and flavonoid glycosides from the *n*-BuOH fraction. The established HPLC method was used to achieve an ideal separation of the peaks of interest (Figure 2). Table 1 presents the retention times for each compound and the correlation coefficient (*r* ^*2*^ *)* values for the linearity of the method. The salicylate-like compounds in PT leaves were generally abundant. Among the six *Populus* samples, salicin (**1**) and populin (**3**) showed the highest concentrations in PT leaves (Table 2), while salireposide (**2**) had the third-highest concentration after the stems of PT and *P. glandulosa*. Additionally, among the flavonoid glycosides, rutin (**6**) and narcissin (**7**) were present in significantly higher concentrations than in other *Populus* species samples. Thermopsoside (**4**) and cynaroside (**5**) had the third and second-highest concentrations, respectively, among the six samples. Cynaroside (**5**) was particularly abundant in the leaves of *P. tremula* var. *glandulosa*.

**TABLE 2:** Compounds **1**–**9** content in *Populus* species.

Compound	Content (mg/g ext.)					
	Leaves			Stems		
	PT	*P. alba*	*P. tremula* var. *glandulosa*	PT	*P. alba*	*P. tremula* var. *glandulosa*
Salicin (**1**)	19.50 ± 0.11^a^	2.02 ± 0.03^c^	17.88 ± 0.06^b^	5.84 ± 0.02^b^	4.98 ± 0.04^c^	10.23 ± 0.03^a^
Salireposide (**2**)	22.92 ± 0.04^a^	1.82 ± 0.06^c^	17.30 ± 0.11^b^	32.43 ± 0.65^b^	5.11 ± 0.05^c^	34.67 ± 0.44^a^
Populin (**3**)	17.67 ± 0.02^a^	tr	4.55 ± 0.03^b^	9.41 ± 0.07^a^	2.50 ± 0.04^c^	7.30 ± 0.06^b^
Thermopsoside (**4**)	1.27 ± 0.01^a^	1.11 ± 0.01^b^	tr	1.86 ± 0.02^b^	tr	6.00 ± 0.11^a^
Cynaroside (**5**)	4.21 ± 0.01^b^	1.01 ± 0.00^c^	17.92 ± 0.19^a^	0.44 ± 0.00^a^	0.15 ± 0.00^b^	0.11 ± 0.00^c^
Rutin (**6**)	15.10 ± 0.01^a^	5.12 ± 0.01^b^	0.32 ± 0.00^c^	tr	0.52 ± 0.00	tr
Narcissin (**7**)	11.97 ± 0.01^a^	7.20 ± 0.05^b^	1.11 ± 0.01^c^	1.57 ± 0.02^a^	0.67 ± 0.01^b^	0.64 ± 0.00^b^
Luteolin 7-*O*-*β*-D-glucuronide butyl ester (**8**)	0.97 ± 0.00	ND	ND	0.12 ± 0.00	ND	ND
Chrysoeriol 7-*O*-*β*-D-glucuronide butyl ester (**9**)	1.09 ± 0.00	ND	ND	0.20 ± 0.00^b^	0.25 ± 0.00^a^	0.13 ± 0.00^c^

tr, trace; ND, not detected. Values are means ± SD.

^a–c^Different letters for the same compound from the same plant part indicate statistically significant differences (*p* < 0.05) according to the Tukey test.

Luteolin 7-*O*-*β*-d-glucuronide butyl ester (**8**) and chrysoeriol 7-*O*-*β*-d-glucuronide butyl ester (**9**) were commonly found in the leaves and stems of PT, with higher concentrations in the leaves. However, their levels were lower compared to other isolated compounds, as these compounds are rarely found in plants. Luteolin 7-*O*-*β*-d-glucuronide butyl ester (**8**) was not identified in any extracts other than those of PT, and chrysoeriol 7-*O*-*β*-d-glucuronide butyl ester (**9**) was identified in little quantities only in the stems of *P. alba* and *P. tremula* var. *glandulosa*. PT, an endemic species in Korea, seems to contain more phenolic and flavonoid glycosides overall than its parent species, *P. alba* and *P. tremula* var. *glandulosa*.

### 3.3 Radical scavenging activities of the extract and four fractions of PT

Subsequently, we investigated whether the PT extract and its four fractions exhibit radical scavenging activity to evaluate their chemical profiling. The DPPH is a violet-colored, stable free radical with strong absorption in EtOH. When DPPH solution is combined with a chemical that may donate a hydrogen atom, it is transformed to a reduced form, and its color is lost (Kedare and Singh, 2011; Sharma and Bhat, 2009). Table 3 shows that the DPPH radical scavenging activity of the EtOH extract and four fractions of PT was confirmed. The EtOH extract and four fractions of PT exhibited concentration-dependent DPPH radical scavenging activity. The EtOAc PT fraction exhibited DPPH radical scavenging activity of 66.98% ± 2.25% at a concentration of 100 μg/mL. Ascorbic acid, commonly used as a positive control for evaluating chemical profiling, has an IC_50_ value of 66.12 μg/mL in DPPH radical scavenging activity (Jadid et al., 2017). Among the EtOH extract and four fractions of PT, the EtOAc PT fraction had the lowest IC_50_ value (58.89 ± 1.51 μg/mL) for DPPH radical scavenging activity.

**TABLE 3:** Radical scavenging activities of PT extracts and fraction.

Scavenging activity	Concentration (μg/mL)	Sample (%)				
		EtOH ext	*n*-Hexane fr	Chloroform fr	Ethyl acetate fr	*n*-Butanol fr
DPPH radical	5	0.46 ± 2.26^d^	-	-	1.07 ± 2.62^d^	-
	25	10.95 ± 1.18^c^	2.25 ± 2.68^c^	1.50 ± 2.26^c^	23.16 ± 1.61^c^	11.34 ± 3.06^c^
	50	18.58 ± 0.80^b^	9.75 ± 1.58^b^	5.57 ± 1.25^b^	42.30 ± 1.03^b^	22.54 ± 1.96^b^
	100	30.73 ± 1.32^a^	16.27 ± 1.01^a^	15.04 ± 0.94^a^	66.98 ± 2.25^a^	41.08 ± 1.48^a^
ABTS^+^ radical	5	9.47 ± 1.49^d^	-	-	7.49 ± 1.16^d^	1.49 ± 2.20^d^
	25	20.86 ± 1.57^c^	-	5.26 ± 0.37^c^	35.87 ± 1.86^c^	20.44 ± 1.79^c^
	50	28.10 ± 2.26^b^	4.43 ± 1.43^b^	12.88 ± 0.93^b^	58.39 ± 1.14^b^	38.21 ± 1.49^b^
	100	48.69 ± 1.43^a^	12.71 ± 0.97^a^	23.91 ± 0.70^a^	87.39 ± 1.11^a^	63.03 ± 1.01^a^
^**·**^OH radical	5	13.66 ± 3.26^d^	20.80 ± 0.97^d^	13.91 ± 1.37^d^	10.09 ± 0.35^d^	13.28 ± 0.47^d^
	25	26.09 ± 3.04^c^	60.55 ± 0.21^c^	59.09 ± 0.11^c^	48.03 ± 2.97^c^	54.14 ± 0.19^c^
	50	38.82 ± 2.74^b^	75.00 ± 0.43^b^	73.40 ± 0.33^b^	71.49 ± 0.88^b^	69.69 ± 0.52^b^
	100	53.73 ± 4.15^a^	79.15 ± 0.20^a^	77.50 ± 0.23^a^	79.63 ± 0.11^a^	79.57 ± 0.24^a^
O_2_^−^ radical	5	-	5.30 ± 0.58^NS^	9.17 ± 0.92	8.00 ± 2.29^c^	6.49 ± 1.23^NS^
	25	-	-	-	7.12 ± 2.40^c^	8.10 ± 3.83
	50	19.16 ± 1.33^b^	1.86 ± 0.77	-	13.42 ± 2.62^b^	8.94 ± 4.41
	100	26.93 ± 1.87^a^	4.94 ± 0.61	-	24.79 ± 2.80^a^	6.04 ± 3.10

Values are presented as means ± SD., fr, fraction.

^a–d^Means are significantly different (*p* < 0.05) based on Duncan’s multiple range test.

The characteristic absorbance of ABTS^+^, a protonated radical, is high at 600 nm and decreases when these radicals are scavenged. Incubation of ABTS with potassium persulfate produces ABTS^+^. The presence of chemical compounds in the tested extracts that inhibit potassium persulfate activity may reduce ABTS^+^ production (Hernández-Rodríguez et al., 2019). Table 3 presents the efficiency of ABTS^+^ radical scavenging activity at varying sample concentrations. The EtOH extract and four fractions of PT exhibited concentration-dependent ABTS^+^ radical scavenging activity. In this investigation, the EtOAc and *n*-BuOH fractions of PT showed enzymatic inhibition percentages of 87.39% ± 1.11% and 63.03% ± 1.01%, respectively, at a concentration of 100 μg/mL. The results of ABTS^+^ radical scavenging activity reflected a broader range of radical scavenging activity than those of DPPH radical scavenging activity (Table 3), as ABTS^+^ can assess the hydrophilic and hydrophobic samples (Krishnaiah et al., 2011). In previous studies, the IC_50_ value of ascorbic acid, commonly used as a positive control, was 50 μg/mL in ABTS^+^ radical scavenging activity assessments (Vidya et al., 2016). Among the PT extract and its fractions, the EtOAc fraction exhibited the lowest IC_50_ value (31.10 ± 0.47 μg/mL) for ABTS^+^ radical scavenging activity.

Photoproduced Fe (II) interacts with H_2_O_2_ to reduce Fe (III) and generate ^•^OH radicals in the photo-Fenton reaction (Lyngsie et al., 2018). ^•^OH radicals are the most reactive and harmful radicals, implicated in a range of human ailments. They can react vigorously, damaging essential cell components, including mitochondria, DNA, and cell membranes (Lipinski, 2011). Consequently, ^•^OH radicals have been associated with various disorders, including aging, obesity, rheumatoid arthritis, and neurological diseases (Pizzino et al., 2017; Martemucci et al., 2022). Therefore, eliminating ^•^OH radicals provides protection against various ailments. Table 3 shows the ^•^OH radical scavenging activity of the EtOH extract and four fractions of PT. Except for the extracts, all four fractions exhibited significantly high radical scavenging activity at concentrations of 5, 25, 50, and 100 μg/mL. The EtOAc fractions of PT demonstrated over 90% radical scavenging activity in all concentrations. These findings indicate that all fractions of PT exhibit potent ^•^OH radical scavenging activity.

NADH phosphate oxidase, a membrane-bound enzyme that reduces free molecular oxygen by one electron, generates O_2_^−^ radicals (Chun et al., 2003). These O2- radicals can further produce additional ROS, such as ^•^OH radicals and H_2_O_2_, leading to oxidative damage in the body (Benov, 2001; Chung and Jeon, 2011). Table 3 presents the O_2_^−^ radical scavenging activity of the EtOH extract and four PT fractions. Furthermore, only the EtOH extract and EtOAc fraction of PT exhibited more than 20% radical scavenging activity at a concentration of 100 μg/mL. While the scavenging activity of the EtOH extracts and EtOAc fractions was relatively high, their overall O_2_^−^ scavenging activity was lower compared to the scavenging activities observed for other radicals such as DPPH, ABTS^+^, and ^•^OH.

In other studies on PT, Kwon et al. (2020) reported higher radical scavenging activity in assessments of DPPH and O_2_^−^ radical scavenging (with IC_50_ values for DPPH, ^•^OH, and O2^−^ of the EtOAc fraction at 8.21, 1.06, and 39.99 μg/mL, respectively). Despite being the same plant species, variations in radical scavenging activity can arise owing to differences in results depending on plant origin, cultivation environment, and experimental conditions. However, our overall results of radical scavenging activities showed a similar trend, with the EtOAc fraction also demonstrating the highest radical scavenging activity. Therefore, our findings indicate that the EtOH extract and four fractions of PT possess radical scavenging activity *in vitro*, scavenging DPPH, ABTS^+^, ^•^OH, and O_2_^−^ radicals. Particularly, the EtOAc fraction exhibited the strongest radical scavenging activity among the extract and other fractions, highlighting it as a potent fraction of PT.

Three compounds such as salicin (**1**), salireposide (**2**), and populin (**3**), were isolated from the EtOAc PT fraction, and their structures were identified (Figure 1). Table 4 presents the DPPH, ABTS^+^, ^•^OH, and O_2_^−^ radical scavenging activities of the isolated compounds from the EtOAc fractions of PT, which were evaluated to assess their chemical profiles. Most radical scavenging activities showed concentration-dependent behavior. Among these three compounds, salireposide (**2**) exhibited the lowest IC_50_ value of 0.33 ± 0.06, 0.19 ± 0.01, 0.02 ± 0.00, and 0.07 ± 0.01 mM for DPPH, ABTS^+^, ^•^OH, and O_2_^−^ radical scavenging activity, respectively. Salireposide (**2**) exhibited the highest scavenging activities among all radical scavenging activities experiments, except for •OH radical scavenging activity. It is reasonable to infer that salireposide (**2**) plays a significant role in contributing to the high radical scavenging ability of the EtOAc fraction of PT.

**TABLE 4:** Radical scavenging activities of active compounds **1**–**3** from PT.

Scavenging activity	Concentration (μg/mL)	Compound (%)		
		Salicin (**1**)	Salireposide (**2**)	Populin (**3**)
DPPH radical	1	-	-	4.42 ± 1.73^b^
	2.5	-	7.99 ± 2.60^c^	6.22 ± 1.75^b^
	5	0.66 ± 0.99^b^	15.68 ± 0.88^b^	9.27 ± 0.70^a^
	10	4.87 ± 1.89^a^	22.63 ± 1.59^a^	2.21 ± 1.95^c^
	IC_50_ (mM)	25.30 ± 12.36	0.33 ± 0.06	-
ABTS^+^ radical	1	-	6.08 ± 0.44^d^	0.03 ± 1.58^b^
	2.5	-	10.82 ± 0.85^c^	1.53 ± 2.01^ab^
	5	1.78 ± 1.30^b^	18.66 ± 0.62^b^	2.36 ± 2.48^ab^
	10	5.35 ± 3.03^a^	29.72 ± 1.19^a^	3.02 ± 2.71^a^
	IC_50_ (mM)	13.54 ± 4.26	0.19 ± 0.01	-
^**·**^OH radical	1	20.82 ± 2.21^d^	26.86 ± 1.00^d^	15.41 ± 2.22^d^
	2.5	39.12 ± 0.77^c^	39.48 ± 1.46^c^	28.32 ± 2.94^c^
	5	49.21 ± 2.21^b^	44.34 ± 1.00^b^	42.29 ± 2.51^b^
	10	53.00 ± 1.42^a^	51.78 ± 0.79^a^	50.90 ± 1.62^a^
	IC_50_ (mM)	0.02 ± 0.00	0.02 ± 0.00	0.02 ± 0.00
O_2_^−^ radical	1	5.22 ± 1.06^d^	13.62 ± 2.03^d^	16.82 ± 1.53^c^
	2.5	18.33 ± 1.10^c^	19.89 ± 3.45^c^	25.77 ± 4.68^b^
	5	27.98 ± 1.64^b^	32.52 ± 4.58^b^	27.91 ± 4.84^b^
	10	33.76 ± 2.04^a^	37.12 ± 1.59^a^	37.52 ± 4.24^a^
	IC_50_ (mM)	0.11 ± 0.01	0.07 ± 0.01	0.12 ± 0.05

Values are means ± SD.

^a–d^Means are significantly different (*p* < 0.05) by Duncan’s multiple range test. IC_50_ is the concentration in μg/mL required to inhibit the formation of radicals by 50%.

In the context of structure-activity relationships, hydroxyl groups typically donate hydrogen atoms and stabilizing free radicals. Hydroxyl groups in the *ortho* or *para* positions (not *meta*) enhance radical scavenging activity by stabilizing the resulting phenoxyl radical through resonance. Compounds including salicin, salireposide, and populin, which form intramolecular hydrogen bonds, exhibit heightened radical scavenging activity owing to phenoxyl radical stabilization (Tirado-Kulieva et al., 2022; Spiegel et al., 2020). Furthermore, glycosylation of phenolic compounds seems to increase their radical scavenging activities potential, possibly by enhancing bioavailability and serving as prodrugs that release more bioactive aglycones *in vivo* upon hydrolysis (Johnson et al., 2021; Zhang et al., 2006). Therefore, this structural characteristic of salicin (**1**), salireposide (**2**), and populin (**3**) significantly contributes to the observed high radical scavenging activity in the EtOAc fraction.

### 3.4 Cytotoxicity of PT extracts and fractions on RAW264.7 cells

**FIGURE 3:**
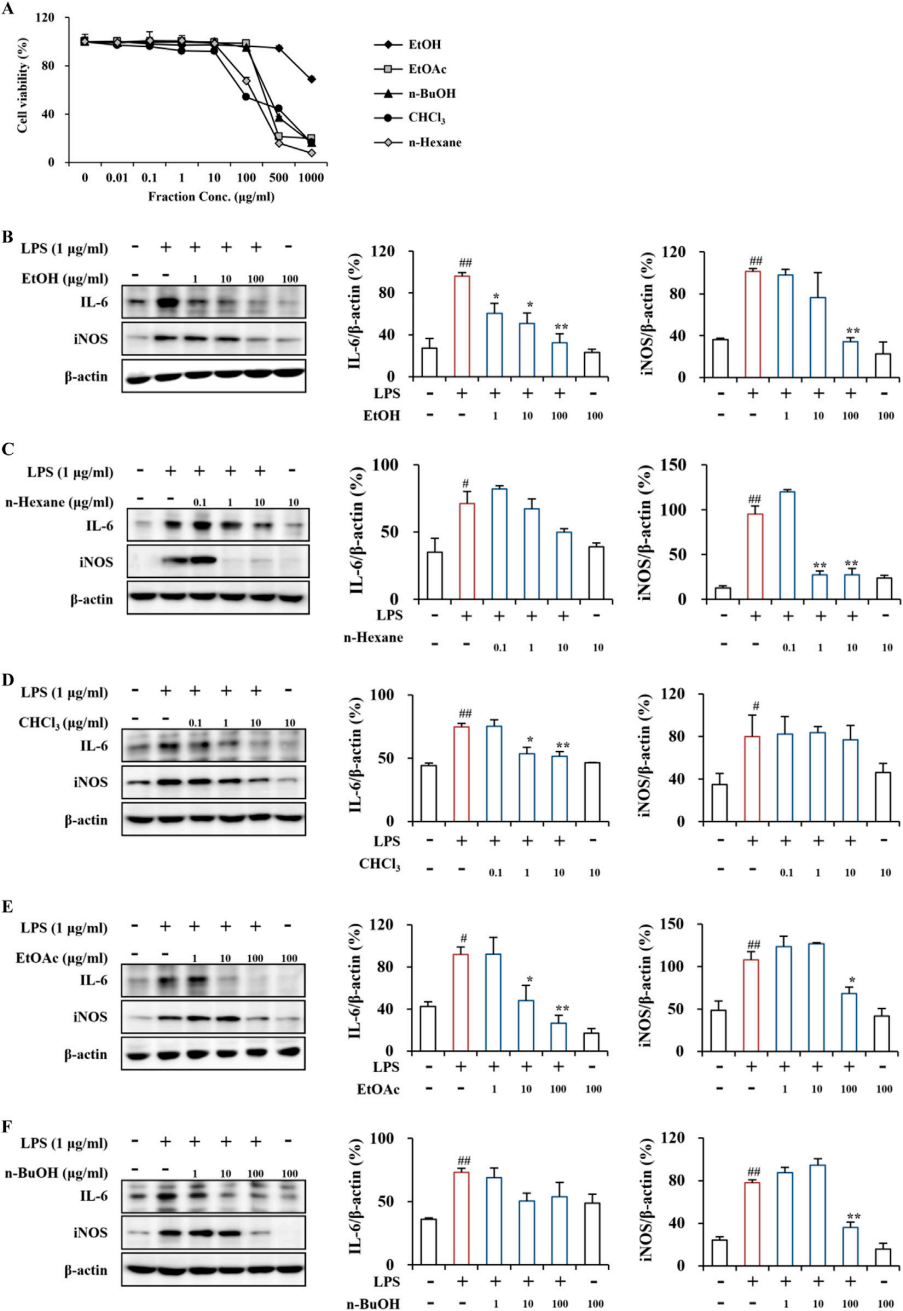
Cytotoxicity and anti-inflammatory effects of PT extract and its fractions on RAW264.7 cells. **(A)** Cytotoxicity of the PT extract and its fractions on RAW264.7 cells. RAW264.7 cells were treated with the PT extract and its four fractions (EtOH, *n*-hexane, CHCl₃, EtOAc, and *n*-BuOH solvents), and cell viability was assessed using an MTT assay. **(B–F)** Anti-inflammatory effects of the PT extract and its fractions on LPS-induced RAW264.7 cells. LPS-induced RAW264.7 cells were pretreated with the PT extract and its four fractions using EtOH **(B)**, *n*-hexane **(C)**, CHCl₃ **(D)**, EtOAc **(E)**, and *n*-BuOH **(F)** solvents. The cell lysates (*n* = 3 per group) from all groups were analyzed by Western blot analysis using IL-6 and iNOS antibodies **(B–F)**, representing the inflammatory cytokine and enzyme, respectively. The results were quantified **(B–F)**, and data are presented as the mean expressive value (the ratio of each value against GAPDH for each sample) ± SEM (one-way ANOVA with *post hoc* test; **p < 0.01 compared to the Control group, *p < 0.05 and **p < 0.01 compared to the LPS-induced group).

To compare the effect of the PT extract and its fractions on the viability of RAW264.7 cells, an MTT assay was performed (Figure 3A). The viability of RAW264.7 cells treated with the PT extract and its four fractions did not significantly differ from the vehicle control (DMSO) at concentrations of 0.01–500 μg/mL for the EtOH extract, 0.01–100 μg/mL for the EtOAc and *n*-BuOH fractions, and 0.01–10 μg/mL for the *n*-hexane and CHCl_3_ fractions (Figure 3A). These results suggest that the PT extract and its fractions do not exhibit specific cell toxicity at concentrations below 500, 100, or 10 μg/mL.

### 3.5 Anti-inflammatory effects of PT extracts and its fractions in LPS-induced RAW264.7 cells

In the LPS-induced RAW264.7 cells, the EtOH extract clearly reduced the protein expression of the representative proinflammatory cytokine IL-6 at concentrations of 1–100 μg/mL and the enzyme iNOS at 100 μg/mL (Figure 3B). The *n*-hexane fraction did not significantly influence IL-6 expression at concentrations of 0.1–10 μg/mL; however, effectively decreased iNOS protein expression at concentration of 1 and 10 μg/mL (Figure 3C). The CHCl₃ fraction significantly downregulated IL-6 protein expression at concentration of 1 and 10 μg/mL; however, did not influence iNOS protein expression at concentrations of 0.1–10 μg/mL (Figure 3D). The EtOAc fraction significantly reduced the protein expression of IL-6 and iNOS at the concentration of 10 and 100 and 100 μg/mL, respectively (Figure 3E). The *n*-BuOH fraction did not significantly affect the protein expression of IL-6 and iNOS at concentrations of 1–100 μg/mL; however, it markedly down-regulated iNOS expression at 100 μg/mL (Figure 3F). Additionally, the EtOH extract and four fractions of PT demonstrated anti-inflammatory activity by inhibiting the protein expression of the representative pro-inflammatory cytokine IL-6 and the enzyme iNOS in the LPS-induced RAW264.7 macrophages (Figure 3). The anti-inflammatory activity of the EtOH extract and EtOAc fraction was superior to that of other fractions (Figure 3). A recent study reported that pretreatment with PT extract mitigated neuronal loss by alleviating glial activation (an indicator of neuroinflammation) in the gerbil hippocampal CA1 area caused by transient global cerebral ischemia (Park et al., 2017). Neuroinflammation, a characteristic feature of neurological disorders, primarily results from chronically activated glial cells (astrocytes and microglia) in the pathological lesion (Kwon and Koh, 2020). Activated glia produces various proinflammatory cytokines, including IL-6 and IL-1β, and enzymes such as iNOS and cyclooxygenase-2 (Kwon and Koh, 2020). Thus, blocking glial activity may be a novel approach to regulating various neurological disorders (Kwon and Koh, 2020). Since non-neurological disorders, including autoimmune and chronic diseases, also involve inflammatory reactions (Laveti et al., 2013), further exploration of PT is necessary to understand its physiological and pathological activities.

## 4 Conclusion

Nine phenolic compounds were isolated from the PT leaves, including novel butyl ester derivatives of luteolin and chrysoeriol, which have not been previously described in the Salicaceae family. The EtOH extract and its fractions showed potent and dose-dependent radical scavenging and anti-inflammatory activities against DPPH, ABTS^+^, hydroxyl radicals, superoxide anions, IL-6, and iNOS. The EtOAc fraction demonstrated superior activity in most assays compared to the extract and other fractions. While salireposide (**2**) demonstrated significant radical scavenging activities capabilities, attributing the efficacy of PT solely to this compound would be premature. The observed bioactivities likely result from the synergistic interaction of various phytochemicals within PT. This study emphasizes the anti-inflammatory potential of PT, suggesting its potential as a valuable source of natural bioactive metabolites for developing functional food and therapeutic agents.

## Data Availability

The original contributions presented in the study are included in the article/supplementary material, further inquiries can be directed to the corresponding authors.
